# Functional and Regulatory Effects of Factor V Leiden and Factor V rs6028 in Breast Cancer

**DOI:** 10.3390/genes16070735

**Published:** 2025-06-25

**Authors:** Sara Marie Lind, Marit Sletten, Carola Elisabeth Henriksson, Mari Tinholt, Nina Iversen

**Affiliations:** 1 Department of Medical Genetics, Oslo University Hospital and University of Oslo, 0450 Oslo, Norway; s.m.lind@studmed.uio.no (S.M.L.); uxarsl@ous-hf.no (M.S.); matinh@ous-hf.no (M.T.); 2 Institute of Clinical Medicine, Faculty of Medicine, University of Oslo, 0450 Oslo, Norway; c.e.henriksson@medisin.uio.no; 3 Department of Medical Biochemistry, Oslo University Hospital, 0450 Oslo, Norway

**Keywords:** Factor V, Factor V Leiden, single nucleotide polymorphisms, breast cancer, gene expression regulation

## Abstract

**Background/Objectives**: Cancer progression and the hemostatic system are closely linked. Coagulation factor V (FV) has a key function in coagulation, with both pro- and anticoagulant functions. FV gene (*F5*) expression and *F5* variants have been linked to breast cancer progression. The direct impact of *F5* variants on FV expression and functional effects in breast cancer are unknown. We aimed to investigate whether the *F5* variants FV Leiden (*F5* rs6025) and *F5* rs6028 influenced FV expression, coagulant activity, and apoptosis in breast cancer cells. **Methods**: MDA-MB-231 cells were transfected with overexpression plasmids containing *F5* wild type, *F5* rs6025 or *F5* rs6028. We investigated the functional impact of the *F5* variants on *F5* mRNA, FV protein, FV coagulant activity, and apoptosis in vitro, and examined the potential of the variants as transcriptional regulators of *F5* expression in silico. **Results**: Increased *F5* mRNA, FV protein, and apoptosis were observed in cells transfected with *F5* wild-type overexpression plasmid compared to empty vector. *F5* mRNA, protein, coagulant activity, and apoptosis were further increased with the *F5* rs6025 and *F5* rs6028 variants compared to *F5* wild type. Cis-expression quantitative trait loci analyses indicated a regulatory effect of *F5* rs6028, and putative transcription factor binding sites for several transcription factors overlapped with the position of *F5* rs6025. **Conclusions**: Our study demonstrated that *F5* rs6025 and *F5* rs6028 have a regulatory effect on FV synthesis that might affect apoptosis in breast cancer. The *F5* variants might therefore enhance the tumor suppressor function of FV in breast cancer.

## 1. Introduction

The close relationship between cancer and the hemostatic system is well established [1]. Cancer patients are at increased risk of developing thrombosis [2]. Moreover, the hemostatic system promotes cancer pathogenesis [1,3]. Coagulation factor V (FV) is a non-enzymatic coagulation factor which is synthesized in the liver and present in blood circulation as an inactive precursor. FV is crucial in secondary hemostasis, exhibiting both pro- and anticoagulant properties. The procoagulant state occurs when activated FV (FVa) acts as a cofactor for coagulation factor X, accelerating thrombin generation. Conversely, acting as an anticoagulant cofactor to activated protein C (APC) and protein S, FV is able to inactivate activated coagulation factor VIII [4]. Alternative roles of FV are emerging. We have reported that FV was elevated in breast cancer tumors, and that high *F5* mRNA levels were associated with improved overall survival and treatment response to chemotherapy, as well as induced apoptosis and reduced proliferation in breast cancer cells [5,6].

Single nucleotide polymorphisms (SNPs) and mutations in the FV gene (*F5*) can influence the properties of FV and lead to thrombosis or bleeding [4]. Factor V Leiden (*F5* rs6025) is an extensively studied point mutation (c.1601G>A; p.Arg534Gln) resulting in the substitution of glutamine with arginine at position 506 of FV [4]. *F5* rs6025 affects a critical cleavage site for APC, leading to APC resistance [4], and weakens the interaction between tissue factor pathway inhibitor α and FV [7]. These mechanisms contribute to an elevated risk of thrombosis, with heterozygous carriers showing an approximately seven-fold increased risk and homozygous carriers an approximately twenty-fold increase [8]. Cancer patients with *F5* rs6025 have a two-fold increased risk of thrombosis compared to cancer patients without *F5* rs6025 [9]. Research exploring associations between *F5* rs6025 and cancer risk has shown diverse results [10,11,12,13,14,15,16]. Of note, we have previously demonstrated an association between four intronic *F5* SNPs and risk of breast cancer [16].

We identified the SNPs in high linkage disequilibrium (LD) with the four target SNPs and found strong LD with the synonymous SNP *F5* rs6028, which was the only SNP in LD that was located in an exon. Although synonymous SNPs do not change the encoded amino acid, they can influence mRNA splicing, stability, and structure, as well as protein folding [17]. This positions *F5* rs6028 as a promising candidate for investigating potential functional effects. To our knowledge, this SNP has not yet been studied in cancer, and the functional impact of *F5* variants in general is scarcely investigated.

In this study, we aimed to gain a better understanding of functional and regulatory effects of *F5* rs6025 and *F5* rs6028 in breast cancer cells.

## 2. Materials and Methods

### 2.1. Cell Culture

The triple-negative human breast epithelial adenocarcinoma cell line MDA-MB-231 (American Type Culture Collection) was cultured in Dulbecco’s Modified Eagle Medium (Gibco, Waltham, MA, USA) containing 4.5 g/L glucose and L-glutamine and 10% fetal bovine serum (Biowest, Nuaillé, France). The cells were maintained at 37 °C in a humidified atmosphere with 5% CO_2_.

### 2.2. Expression Vector and Mutagenesis

The pMT2-FV wild type (pFV-wt) [5] was used as a template to create plasmids with *F5* rs6025 or *F5* rs6028 using the QuikChange II XL Site-Directed Mutagenesis Kit (Agilent Technologies, Santa Clara, CA, USA). *F5* rs6025 was introduced in a plasmid named pMT2-FV-rs6025 (pFV-rs6025) with the following primers: forward 5′-GCAGATCCCTGGACAGGCAAGGAATACAGAGGGCAGC-3′ and reverse 5′-GCTGCCCTCTGTATTCCTTGCCTGTCCAGGGATCTGC-3′. *F5* rs6028 was introduced in a plasmid named pMT2-FV-rs6028 (pFV-rs6028) with the following primers: forward 5′-GAAAAACCACAGTCTACCATTTCAGGACTTCTTGGGCC-3′ and reverse 5′-GGCCCAAGAAGTCCTGAAATGGTAGACTGTGGTTTTTC-3′. The mutagenesis was verified with Sanger sequencing.

### 2.3. Transient Transfection and Harvesting of mRNA and Protein Lysates

MDA-MB-231 cells seeded in 6-well plates were transiently transfected using Lipofectamine™ 3000 (Thermo Fisher Scientific, Waltham, MA, USA) with 2.5 µg pMT2 empty vector, pFV-wt, pFV-rs6025, or pFV-rs6028. Transfection efficiency for the plasmids was tested by analyzing green fluorescent protein and tissue factor pathway inhibitor mRNA expression. Cells and media were harvested 48 h post transfection. Total RNA was isolated from cell lysates using the RNAqueous Total RNA Isolation Kit (Thermo Fisher Scientific). Cell lysates for protein analyses were harvested using RIPA buffer (Sigma-Aldrich, St. Louis, MO, USA) with 1xHalt Proteinase and Phosphatase Inhibitor (Thermo Fisher Scientific).

### 2.4. Quantitative Measurement of F5 mRNA

Total RNA was quantified with NanoDrop^®^ND-1000 and cDNA was synthesized using the High-Capacity cDNA Reverse Transcription Kit (Thermo Fisher Scientific). Quantification of mRNA was performed with quantitative RT-PCR (qPCR) using the QuantStudio 12k Flex Real-Time system with the TaqMan Gene Expression Master Mix (Thermo Fisher Scientific) and TaqMan assays for *F5* (Hs00914120_m1; Thermo Fisher Scientific) and Phosphomannomutase 1 (*PMM1*; in house) as endogenous control. Relative mRNA quantification (RQ) values for the *F5* variants compared to pFV-wt was calculated using the comparative CT (ΔΔCT) method.

### 2.5. Measurement of FV Protein and FV Coagulant Activity

In cell media, FV protein was measured using ZYMUTEST Factor V (Hyphen-BioMed, Neuville-sur-Oise, France). The absorbance was measured with a VersaMax microplate reader (Emerson, St. Louis, MO, USA), and the SoftMax Pro 6.4 software was used to calculate protein concentrations. FV protein values in cell media were corrected for total protein concentrations in cell lysates, which was measured using the PIERCE BCA Protein Assay Kit (Thermo Fisher Scientific). In cell media, measurement of FV coagulant activity was performed as a one-stage clotting assay with prothrombin RecombiPlasTin^®^2G (Werfen, Barcelona, Spain) as initiating reagent on an ACL TOP 700 (Instrumentation Laboratory, Bedford, MA, USA). The values for the *F5* variants were calculated relative to pFV-wt.

### 2.6. Detection of Apoptosis

Apoptosis was determined by quantification of nucleosomes in fresh cell lysates using the Cell Death Detection ELISA^PLUS^ kit (Roche Applied Science, Penzberg, Germany). The absorbance was measured using a VersaMax microplate reader. The values for the *F5* variants were calculated relative to pFV-wt.

### 2.7. In Silico Analyses

Putative transcription factor binding sites overlapping with the positions of the *F5* variants were studied using Remap 2022 [18] in the UCSC Genome Browser [19]. Remap provides data from ChIP-Seq experiments. Expression quantitative trait locus (eQTL) analysis (±1M base pair) was performed using FIVEx [20]. FIVEx is an interactive eQTL browser comprising the Gene-Tissue Expression (GTEx) data for visualization and functional interpretation of gene variants. FIVEx offers an intuitive interface that visualizes *p*-values alongside negative and positive effect sizes, representing decreased and increased expression for the alternative allele, respectively.

### 2.8. Statistical Analyses

Normality of the data was tested using the Shapiro–Wilks test. Statistics were calculated using the Kruskal–Wallis test followed by the Wilcoxon rank sum test. *p*-values < 0.05 were considered statistically significant.

## 3. Results and Discussion

*F5* expression and *F5* variants have been linked to breast cancer pathogenesis [6]. However, conflicting results are reported for the association between *F5* rs6025 and cancer risk. Vossen et al. reported that homozygous carriers of *F5* rs6025 showed an increased risk of colorectal cancer, whereas heterozygous carriers showed a decreased risk [10]. However, several studies with *F5* rs6025 heterozygotes have failed to find associations to colorectal, gastric, gastrointestinal, oral, and gynecological cancers [11,12,13,14,15]. Similarly, we were not able to find an association between *F5* rs6025 heterozygotes and breast cancer risk [16]. It is challenging to perform studies with sufficient statistical power when the frequency of a variant is low. Of note, only about 0.02% of the Caucasian population is *F5* rs6025 homozygotes [21]. In vitro studies in cell lines are an alternative strategy to study direct effects of *F5* variants in cancer. In this study, we aimed to explore functional and potential regulatory effects of the *F5* rs6025 and *F5* rs6028 variants in breast cancer.

### 3.1. Functional Effects of F5 Variants on FV Production, Secretion, and Coagulant Activity

**Figure 1:**
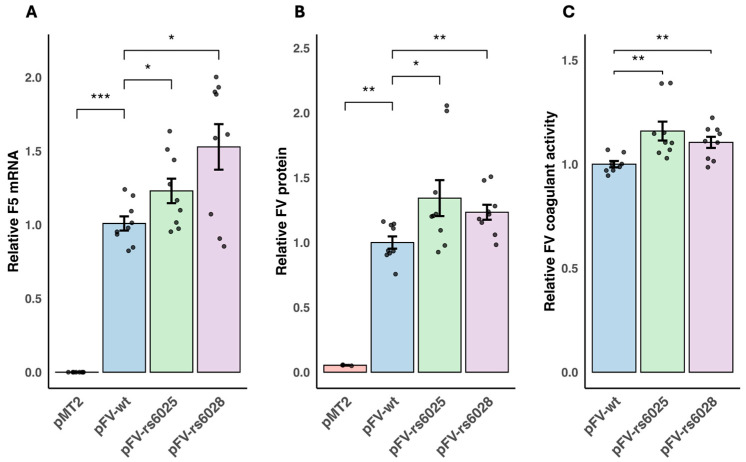
Functional effects of *F5* variants on *F5* mRNA, FV protein, and FV coagulant activity. (**A**) *F5* mRNA expression and (**B**) FV protein levels in MDA-MB-231 cells transfected with pMT2, pFV-wt, pFV-rs6025, or pFV-rs6028 for 48 h. (**C**) FV coagulant activity in MDA-MB-231 cells transfected with pFV-wt, pFV-rs6025, or pFV-rs6028 for 48 h. mRNA expression was measured with qPCR and normalized against endogenous control. FV protein levels in media were measured with FV ELISA and corrected for the total protein concentration in cell lysates. FV coagulant activity was measured in cell media with one-stage assay. *F5* mRNA, FV protein, and FV coagulant activity were calculated relative to pFV-wt. Mean values and SEM were calculated from three independent experiments. Significant differences were calculated using the Kruskal–Wallis test followed by the Wilcoxon rank sum test and marked with * (*p* < 0.05), ** (*p* < 0.01), *** (*p* < 0.001).

**Figure A1:**
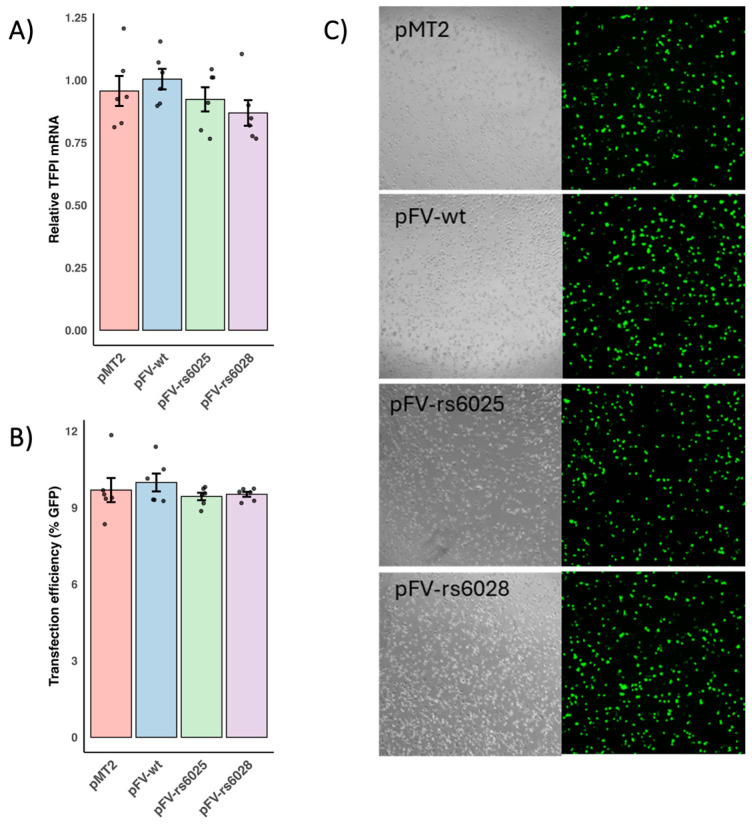
Transfection efficiency. The wild-type factor V plasmid, pMT2-FV-wt, the empty vector pMT2, and the *F5* variant plasmids pFV-rs6025 and pFV-rs6028 were each co-transfected into MDA-MB-231 cells with Lipofectamine3000 (Thermo Scientific, Waltham, MA, USA). (**A**) Co-transfection with an equal amount of a plasmid expressing tissue factor pathway inhibitor (*TFPI*) (pTOPO-TFPIα). After 24 h of incubation, cells were harvested, RNA was isolated from cell lysates with the RNAqueous™ Total RNA Isolation Kit (Thermo Fisher Scientific), and cDNA synthesized using the High-Capacity cDNA Reverse Transcription Kit (Thermo Fisher Scientific). Quantitative RT-PCR (qPCR) was performed with a *TFPI*-specific TaqMan assay and TaqMan Gene Expression Master Mix (Thermo Fisher Scientific). All samples were run in triplicate on the Applied Biosystems QuantStudio 12K Flex Real-Time System (Thermo Scientific). Relative *TFPI* mRNA expression was calculated using the comparative CT (ΔΔCT) method with hypoxanthine phosphoribosyltransferase 1 (*HPRT1)* as the endogenous control. *TFPI* mRNA expression in wells co-transfected with the variant FV plasmids was calculated as fold change relative to *TFPI* mRNA expressed in the wells co-transfected with the wild-type FV plasmid. Mean results and SEM from two independent experiments (*n* = 6) are shown. (**B**) Co-transfection with an equal amount of a plasmid expressing green fluorescent protein (GFP), pmaxGFPTM Vector (Lonza, Slough, UK). Twenty-four hours post transfection, cells were examined for GFP fluorescence in a Nikon ECLIPSE Ts2-FL microscope (Nikon, Tokyo, Japan) and cells counted and transfection efficiency calculated using ImageJ 1.54g. Mean results and SEM from two independent experiments (*n* = 6) are shown. (**C**) Pictures from one representative experiment are shown.

We studied the effects of the *F5* variants on *F5* mRNA, FV protein, and FV coagulant activity. Transfection efficiency was equal for all plasmids (Figure A1). *F5* mRNA and protein were significantly elevated in cells transfected with pFV-wt compared to the empty vector pMT2 (*P* = 8.2 × 10^−5^ and *P* = 9.1 × 10^−3^, respectively). Compared to pFV-wt, we observed a 23% and 53% increase in *F5* mRNA levels for the pFV-rs6025 (*P* = 4.0 × 10^−2^) and pFV-rs6028 (*P* = 4.0 × 10^−2^) transfected cells, respectively (Figure 1A). Supporting this, FV protein levels in cell media were increased by 34% and 23% for pFV-rs6025 (*P* = 1.9 × 10^−2^) and pFV-rs6028 (*P* = 4.0 × 10^−3^), respectively (Figure 1B). These results showed that the elevated *F5* mRNA expression for the *F5* variants resulted in increased secretion of FV to cell media. Furthermore, coagulant activity of FV in cell media was elevated by 16% and 10% in cells transfected with pFV-rs6025 (*P* = 2.8 × 10^−3^) and pFV-rs6028 (*P* = 9.2 × 10^−3^), respectively, compared to pFV-wt (Figure 1C), which show that the increase in secreted FV protein was coagulant active in both variants. Supporting this result, we have, of note, observed elevated plasma D-dimer levels in *F5* rs6025 carriers compared to non-carriers in breast cancer patients, which indicated increased coagulant activity in *F5* rs6025 carriers with breast cancer. Differences in the transfection efficiency could correlate with increased mRNA and protein expression observed for the *F5* variants compared to *F5* wild type. We conducted control experiments with co-transfection of the *F5* variant plasmids and a control plasmid with equal amounts of either GFP or *TFPI* plasmids. The results reflected equal transfection efficiency across the *F5* plasmids (Figure A1). We can then assume that the differences we observed between the *F5* variants are not because of differences in transfection efficiency, but specifically because of the *F5* variants.

### 3.2. Functional Effects of F5 Variants on Apoptosis

**Figure 2:**
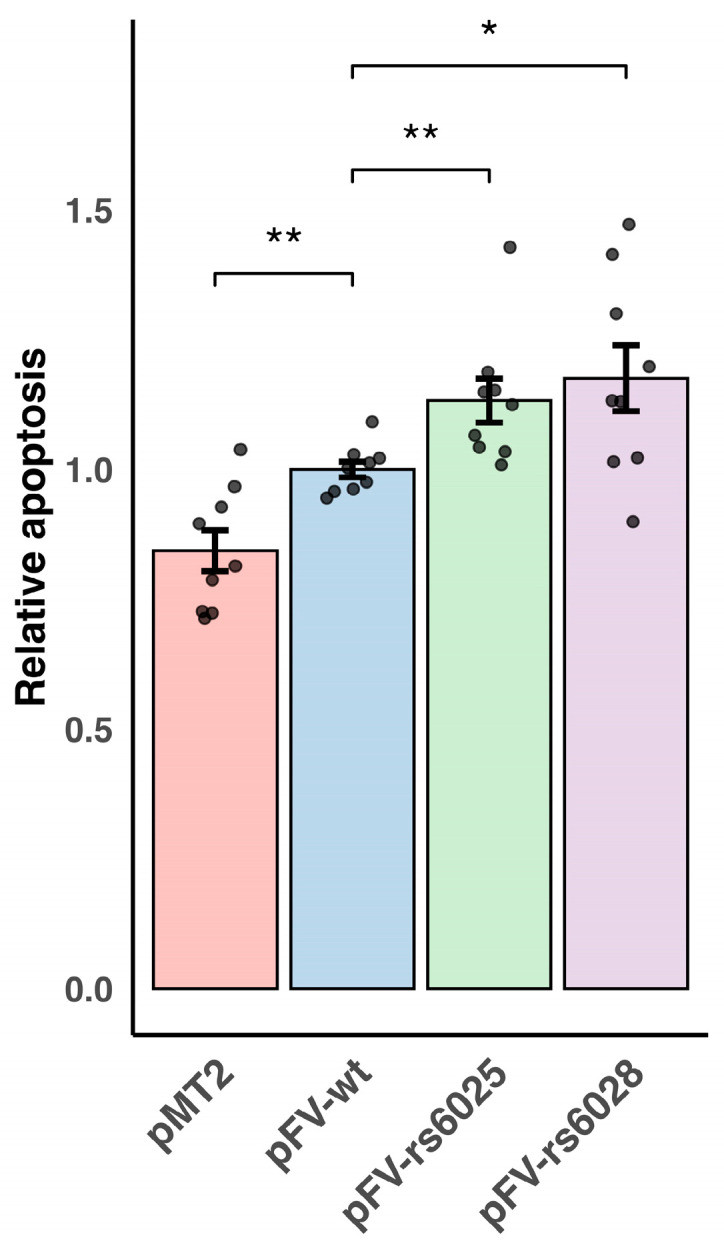
Functional effects of *F5* variants on apoptosis. MDA-MB-231 cells were transfected with pMT2, pFV-wt, pFV-rs6025, or pFV-rs6028. Apoptosis was measured by detection of nucleosomes 48 h post transfection. The values for the *F5* variants were calculated relative to pFV-wt. Mean values and SEM were calculated from three independent experiments. Significant differences were calculated using the Kruskal–Wallis test followed by the Wilcoxon rank sum test and marked with * (*p* < 0.05), ** (*p* < 0.01).

Apoptosis was significantly elevated in cells transfected with pFV-wt compared to the empty vector pMT2 (*P* = 7.8 × 10^−3^). Compared to pFV-wt, we observed a modest 13% increase in apoptosis in cells transfected with pFV-rs6025 (*P* = 1.9 × 10^−3^) and an 18% increase in cells transfected with pFV-rs6028 (*P* = 2.4 × 10^−2^) (Figure 2). We have previously reported that overexpression of the FV wild type induced apoptosis in MDA-MB-231 cells [5]. The increased effect on apoptosis observed in cells transfected with pFV-rs6025 and pFV-rs6028, compared to pFV-wt, may therefore be due to the higher levels of FV per se, rather than a novel function caused by the *F5* variants. The results show that the *F5* variants enhances tumor suppressor functions, possibly via increasing the FV expression, which might be relevant in breast cancer pathology. Interestingly, we have previously reported that *F5* rs9332542, the SNP in strong LD with *F5* rs6028, was associated with decreased risk of breast cancer which might support these results. While this study focused on a triple-negative cell line, the *F5* variants may exert different apoptotic effects in other breast cancer cell types, such as ER-positive tumors, which typically express lower levels of FV. Further research is needed to determine whether these effects vary across different breast cancer subtypes.

### 3.3. Regulatory Annotation Analysis of the F5 Variants

**Figure 3:**
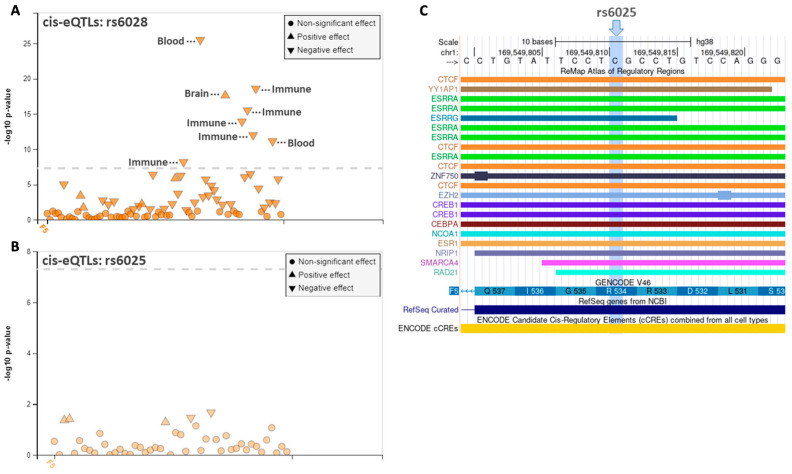
Regulatory effects of *F5* variants. (**A**) Cis-eQTL analysis of *F5* rs6028 and (**B**) *F5* rs6025 using the FIVEx tool [20]. The dashed line illustrates the significance threshold. (**C**) ChIP-Seq peaks in the position for *F5* rs6025 (highligted) using Remap [18] in the UCSC Genome Browser [19]. Abbreviations: ESR1, Estrogen Receptor 1; CTCF, CCCTC-binding factor; YY1AP1, YY1 Associated Protein 1; ESRRA, Estrogen Related Receptor Alpha; ESRRG, Estrogen Related Receptor Gamma; ZNF750, Zinc Finger Protein 750; EZH2, Enhancer Of Zeste 2 Polycomb Repressive Complex 2 Subunit; CREB1, CAMP Responsive Element Binding Protein 1; CEBPA, CCAAT Enhancer Binding Protein Alpha; NCOA1, Nuclear Receptor Coactivator 1; NRIP1, Nuclear Receptor Interacting Protein 1; SMARCA4, SWI/SNF Related Matrix Associated Actin Dependent Regulator Of Chromatin Subfamily A Member 4; RAD21, RAD21 Cohesin Complex Component; NCBI, The National Center for Biotechnology Information.

Considering the elevated expression observed for the *F5* variants compared to pFV-wt, we explored the possible regulatory effect of the *F5* variants by analyzing the associations between the *F5* variants and known eQTLs. We searched for eQTLs located near the gene of origin (*cis*-eQTLs) using the FIVEx tool [20]. *F5* rs6028 was identified as a *cis*-eQTL for *F5* expression with the minor allele having an opposite effect on the gene expression in brain and blood/immune cells, which might indicate a tissue specific effect of the SNP (Figure 3A). The *cis*-eQTL associations for *F5* rs6025 and *F5* expression were not significant after adjusting for multiple testing (Figure 3B).

The functional annotation analyses indicated that *F5* rs6028 may act directly or indirectly as a transcriptional regulator for *F5* in several tissues. This might explain the increased FV expression observed for pFV-rs6028 compared to pFV-wt. Supporting this, we have previously reported that *F5* rs9332542, the SNP in strong LD with *F5* rs6028, was correlated to FV mRNA and protein expression in plasma from breast cancer patients [6].

Since we observed a differential *F5* expression for the *F5* variants relative to the wild type, our hypothesis is that these variants might be located in transcription factor binding sites. This might influence putative binding sites for transcription which potentially can influence functional effects on *F5* expression and apoptosis. To explore this, we used data from ChIP-Seq experimental data from Remap [18]. No ChIP-Seq peaks were observed for the location of *F5* rs6028. In the location of *F5* rs6025, the most profound ChIP-Seq peaks were for the transcription factors Estrogen Related Receptor Alpha (*ESRRA*) (*n* = 5) and CCCTC-binding factor (*CTCF)* (*n* = 4) (Figure 3C). The ChIP-Seq peaks for *ESRRA* were observed in three different breast cancer cell lines and lymphoblasts, whereas the ChIP-Seq peaks for *CTCF* were observed in B-cells, keratinocytes, cardiac cells, and epithelial cells. Stein et al. have reported that Estrogen-related receptor alpha (ERRα) (encoded by the *ESRRA* gene) is critical for growth and migration of estrogen receptor-negative cells [22], and it has been reported that *CTCF* upregulation reduces apoptosis in breast cancer cells [23,24]. The direct effects of *F5* rs6025 on these putative transcription factor binding sites remain to be proven. However, the results suggest that *F5* rs6025 might play a role in transcription regulation, potentially influencing processes like cell growth, migration, or apoptosis.

In this study, we used transient transfection of breast cancer cell lines. Using this approach, we can study a high overexpression of the different variants we want to study, but we can only study short-time effects. The efficiency of transfection between the different variant plasmids may influence the measured outcomes. We conducted co-transfection experiments with the *F5* variant plasmids together with GFP and *TFPI* plasmids, and the results from these experiments show no indirect differences in the efficiency between the *F5* variant plasmids, although an absolute normalization of the transfection efficiency could not be obtained by this method.

Collectively, our study demonstrated functional and regulatory effects of *F5* rs6025 and *F5* rs6028 in breast cancer cells. Our findings suggest that these *F5* variants may enhance the tumor suppressor functions in breast cancer, potentially due to increased FV expression.

## Data Availability

The in silico data presented in this study are openly available in the Remap [18] resource and the FIVEx [20] analysis tool. The original contributions presented in this study are included in the article. Further inquiries can be directed to the corresponding author.

## Abbreviations

The following abbreviations are used in this manuscript:

APC	Activated protein C
eQTL	Expression quantitative trait locus
*F5*	Coagulation factor V gene
*F5* rs6025	FV Leiden
FV	Coagulation factor V
FVa	Activated coagulation factor V
GTEx	Gene–tissue expression
LD	Linkage disequilibrium
PMM1	Phosphomannomutase 1
pFV-rs6025	pMT2-FV-rs6025
pFV-rs6028	pMT2-FV-rs6028
qPCR	Quantitative RT-PCR
RQ	Relative mRNA quantification
SNPs	Single nucleotide polymorphisms
wt	Wild type
ΔΔCT	Comparative CT method
ESR1	Estrogen receptor 1
CTCF	CCCTC-binding factor
YY1AP1	YY1 Associated protein 1
ESRRA	Estrogen-related receptor alpha
ESRRG	Estrogen-related receptor gamma
ZNF750	Zinc finger protein 750
EZH2	Enhancer of zeste 2 polycomb repressive complex 2 subunit
CREB1	CAMP responsive element binding protein 1
CEBPA	CCAAT enhancer binding protein alpha
NCOA1	Nuclear receptor coactivator 1
NRIP1	Nuclear receptor interacting protein 1
SMARCA4	SWI/SNF-related matrix-associated actin-dependent regulator of chromatin subfamily A member 4
RAD21	RAD21 cohesin complex component
NCBI	The national center for biotechnology information
